# The prognostic implication of intraductal carcinoma of the prostate in metastatic castration-resistant prostate cancer and its potential predictive value in those treated with docetaxel or abiraterone as first-line therapy

**DOI:** 10.18632/oncotarget.19520

**Published:** 2017-07-24

**Authors:** Jinge Zhao, Pengfei Shen, Guangxi Sun, Ni Chen, Jiandong Liu, Xin Tang, Rui Huang, Diming Cai, Jing Gong, Xingming Zhang, Zhibin Chen, Xiang Li, Qiang Wei, Peng Zhang, Zhenhua Liu, Jiyan Liu, Hao Zeng

**Affiliations:** ^1^ Department of Urology, Institute of Urology, West China Hospital, Sichuan University, Chengdu, 610041, China; ^2^ Department of Pathology, West China Hospital, Sichuan University, Chengdu, 610041, China; ^3^ Department of Oncology, West China Hospital, Sichuan University, Chengdu, 610041, China; ^4^ Department of Nuclear Medicine, West China Hospital, Sichuan University, Chengdu, 610041, China; ^5^ Department of Ultrasound, West China Hospital, Sichuan University, Chengdu, 610041, China

**Keywords:** metastatic castration-resistant prostate cancer, intraductal carcinoma of prostate, docetaxel, abiraterone, prognosis

## Abstract

Intraductal carcinoma of the prostate (IDC-P) is recognized as a newly pathological entity in 2016 WHO classification. It's role in metastatic castration-resistant prostate cancer (CRPC) remains obscure. We aimed to explore the association of IDC-P with clinical outcome and to further identify its potential predictive role in making first-line treatment decisions for mCRPC. We retrospectively analyzed data of 131 mCRPC patients. IDC-P was diagnosed by re-biopsy at the time of mCRPC. Among total patients, 45 and 41 received abiraterone or docetaxel as first-line therapies, respectively. PSA response, PSA progression-free survival (PSA-PFS) and overall survival (OS) from mCRPC to death were analyzed using Kaplan-Meier curves, Log-rank test, Cox regression models and Harrell's C-index. The incidence of IDC-P in mCRPC reached 47.3%. IDC-P was not only related to rapid PSA progression, but also associated with a 20-month decrease in OS. Among IDC-P(-) patients, PSA response, PSA-PFS and OS were comparable in abiraterone-treated and docetaxel-treated groups. In contrast, among IDC-P(+) patients, PSA response rate is higher in abiraterone-treated group vs. docetaxel-treated group (52.4% vs. 21.7%; *p* = 0.035). Also, PSA-PFS and OS were much longer in the IDC-P(+) abiraterone-treated group vs. the docetaxel-treated group (PSA-PFS: 13.5 vs.6.0 months, *p* = 0.012; OS: not reach vs.14.7 months, *p* = 0.128). Overall, IDC-P in mCRPC from re-biopsy was an independent prognosticator for clinical outcome. Abiraterone was observed having a better therapeutic efficacy than docetaxel as the first-line therapy in IDC-P(+) mCRPC patients. Thus, we suggest IDC-P should be considered as a novel predictive marker helping physicians making treatment decisions for mCRPC.

## INTRODUCTION

To date, there are at least six FDA approved therapeutic agents for metastatic castration-resistant prostate cancer (mCRPC) [1]. Among them, Doc-based chemotherapy and androgen receptor (AR)-directed agents are widely used as first-line therapies [2–6]. However, after an initial response, drug resistance inevitably occurs. The exact mechanisms of drug resistance remain poorly understood [7, 8]. Additionally, optimal therapeutic sequencing strategies for mCRPC are unknown and treatment-guiding markers that could eventually improve this plight are still lacking.

Few biomarkers, such as androgen receptor variant 7 (AR-V7), are considered as predictive markers for optimizing therapeutic schemes for improving treatment selection for patients [9, 10]. However, even the detection of AR-V7 has limitations [11].

Intraductal carcinoma of the prostate (IDC-P) was usually concomitant with acinar adenocarcinoma in prostate cancer (PCa) patients [12]. Due to its morphological features and highly aggressive behavior, IDC-P has been recently recognized as a new pathological entity in the 2016 WHO classification [13]. In our previous studies, IDC-P at initial diagnosis of metastatic PCa was found to be independently associated with shorter time to mCRPC and poorer overall survival (OS) [14]. Among mCRPC patients, incidence of IDC-P obtained by re-biopsy was increased, and patients with IDC-P were clearly associated with rapid disease progression [15]. More interestingly, we also found that IDC-P(+) mCRPC patients were unlikely to be sensitive to docetaxel (Doc)-based chemotherapy [15].

Taken together, it appears that IDC-P is a culprit for the progression of PCa. Thus, agents appropriate for this entity could improve outcomes of mCRPC patient. To test this hypothesis, the aims of this study were to explore the association of IDC-P with clinical outcomes from the time of mCRPC, and further to compare treatment efficacy of Doc or Abi in patients with or without IDC-P.

## RESULTS

### Patient characteristics

Baseline characteristics of the 131 mCRPC patients were summarized in Table 1. The median follow-up time was about 59 months. Nearly half patients (45.8%, 60/131) died at the end of the cut-off point day. The median OS (from mCRPC to death) was 26.0 months. Of note, compared with initial biopsy, IDC-P at re-biopsy nearly doubled from 27.5% (36/131) to 47.3% (62/131). According to the results from re-biopsy, patients were subsequently classified into two groups: men with IDC-P (*n* = 62) and without IDC-P (*n* = 69).

**Table 1:** Baseline characteristics of patients at the time of mCRPC

Variables	Without IDC-P (*n* = 69)	With IDC-P (*n* = 62)	Total (*n* = 131)	*p* value
Age (y)				
Median (IQR)	73.0 (67.0–77.0)	70.0 (62.0–75.0)	72.0 (64.0–75.0)	
≥ 70	45 (65.2%)	32 (51.6%)	77 (58.8%)	0.114
< 70	24 (34.8)	30 (48.4%)	54 (41.2%)	
CRPC-free survival(CFS) (mo)				
Median (IQR)	16.6 (8.8–28.7)	11.1 (6.0–20.5)	13.7 (6.4–24.6)	
≥ 10	51 (73.9%)	35 (56.5%)	86 (65.6%)	0.036
< 10	18 (26.1%)	27 (43.5%)	45 (34.4%)	
1st -line therapy for CRPC, no (%)				
Abiraterone	34 (49.3%)	21 (33.9%)	55 (42.0%)	0.188
Docetaxel	18 (26.1%)	23 (37.1%)	41 (31.3%)	
BSC and others	17 (24.6%)	18 (29.0%)	35 (26.7%)	
Gleason score, no (%)*				
< 8	17 (24.6%)	4 (6.5%)	21 (16.0%)	0.005
8–10	52 (75.4%)	58 (93.5%)	110 (84.0%)	
Castration type, no (%)				
Surgical castration	26 (37.7%)	24 (38.7%)	50 (38.2%)	0.904
Medical castration	43 (62.3%)	38 (61.3%)	81 (61.8%)	
Visceral Metastasis, no (%)				
Without	66 (95.7%)	60 (96.8%)	126 (96.2%)	1.000
With	3 (4.3%)	2 (1.5%)	5 (3.8%)	
ECOG score, no (%)				
0–1	53 (76.8%)	47 (75.8%)	100 (76.3%)	0.892
≥ 2	16 (23.2%)	15 (24.2%)	31 (23.7%)	
Pain score, no (%)				
≥ 3, no (%)	23 (33.3%)	22 (35.5%)	45 (34.4%)	0.796
< 3, no (%)	46 (66.7%)	40 (64.5%)	86 (65.6%)	
Bone Scan Lesions, no (%)				
≥ 10 sites	43 (62.3%)	41 (66.1%)	84 (64.1%)	0.650
< 10 sites	26 (37.7%)	21 (33.9%)	47 (35.9%)	
PSA (ng/ml)				
Median (IQR)	69.3 (14.8–238.4)	62.3 (33.6–122.1)	65.7 (23.3–172.7)	
≥ 100, no (%)	31 (44.9%)	24 (38.7%)	55 (42.0%)	0.472
< 100, no (%)	38 (55.1%)	38 (61.3%)	76 (58.0%)	
PSADT (days)				
Median (IQR)	47.1 (31.8–66.3)	39.9 (26.3–51.7)	44.7 (28.5–58.0)	
≥ 30, no (%)	56 (81.2%)	39 (62.9%)	95 (72.5%)	0.019
< 30, no (%)	13 (18.8%)	23 (37.1%)	36 (27.5%)	
Testosterone (ng/ml)				
Median (IQR)	0.07 (0.02–0.09)	0.09(0.04–0.11)	0.09 (0.02–0.09)	
≥ 0.09, no (%)	31 (44.9%)	40 (64.5%)	71 (54.2%)	0.025
< 0.09, no (%)	38 (55.1%)	22 (35.5%)	60 (45.8%)	
HGB (g/L)				
Median (IQR)	125.0 (118.0–132.0)	125.0(119.0–138.0)	125.0 (119.0–134.0)	
≥ 120, no (%)	52 (75.4%)	46 (74.2%)	98 (74.8%)	0.878
< 120, no (%)	17 (24.6%)	16 (25.8%)	33 (25.2%)	
LDH (IU/L)				
Median (IQR)	256.0 (184.0–308.0)	301.5(208.3–308.0)	263.0 (194.0–308.0)	
≥ 250, no (%)	36 (52.2%)	35 (56.5%)	71 (54.2%)	0.624
< 250, no (%)	33 (47.8%)	27 (43.5%)	60 (45.8%)	
ALP (IU/L)				
Median (IQR)	140.0 (91.5–211.5)	211.5(77.5–211.5)	149.0 (87.0–211.5)	
≥ 160, no (%)	28 (40.6%)	30 (48.4%)	58 (44.3%)	0.369
< 160, no (%)	41 (59.4%)	32 (51.6%)	73 (55.7%)	

*Owing to the endocrine response caused by androgen deprivation therapy, Gleason score at re-biopsy could not be evaluated accurately. Therefore, Gleason score at the initial biopsy instead of the re-biopsy was given in the table.

IQR = interquartile range; CRPC = castration-resistant prostate cancer; CFS = CRPC free survival; IDC-P = intraductal carcinoma of the prostate; BSC = best supportive care; ECOG = Eastern Cooperative Oncology Group; PSA = prostate specific antigen; PSADT = prostate specific antigen doubling time; HGB = hemoglobin; LDH = lactate dehydrogenase; ALP = alkaline phosphatase.

### IDC-P was a strong prognosticator in mCRPC

**Figure 1:**
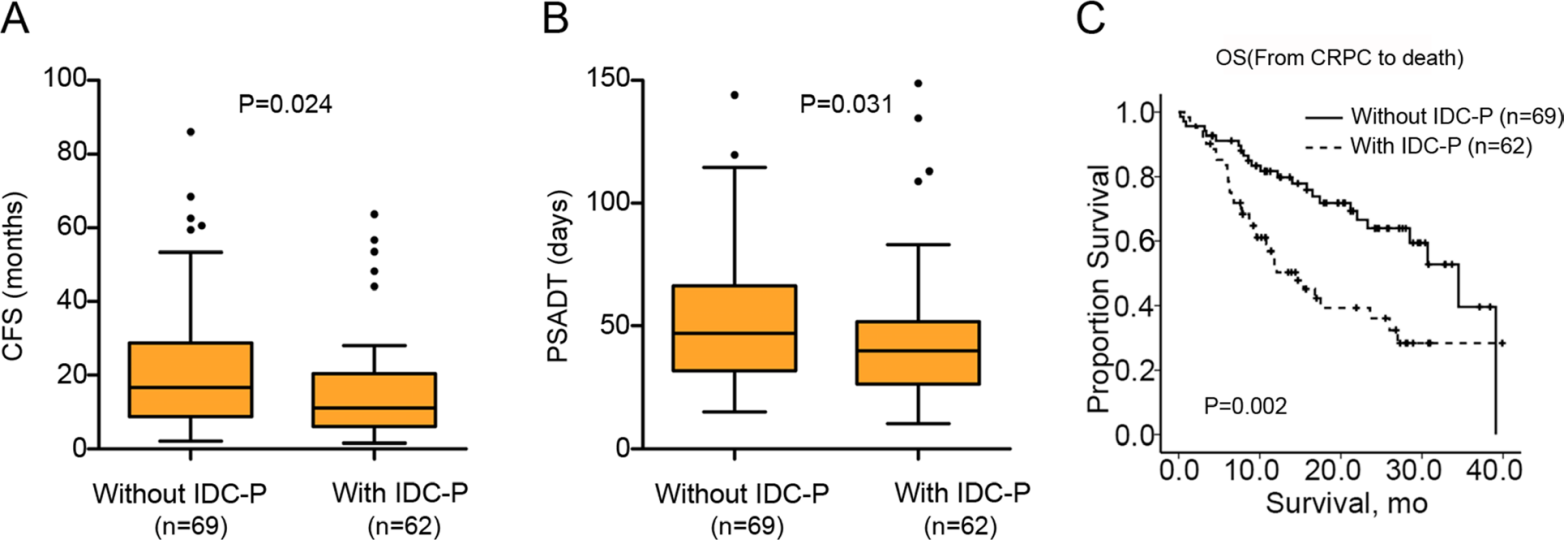
Differences of CFS, PSADT and OS between IDC-P(+) and IDC-P(–) patients (**A**) Box plots of CFS between patients with and without IDC-P; (**B**) Box plots of PSADT between patients with and without IDC-P; (**C**) Kaplan-Meier curves of OS between patients with and without IDC-P.

IDC-P was observed to be associated with relatively shorter CFS (median CFS: 9.9 vs.15.1months, *p* = 0.024, Figure 1A). Compared to men without IDC-P, men with IDC-P had a more rapid PSADT (median time: 39.9 vs.47.1days, *p* = 0.031, Figure 1B), and larger proportion with PSADT less than 30 days (23/62, 37.1% vs. 13/69, 18.8%, *p* = 0.019). Furthermore, the presence of IDC-P significantly reduced OS by nearly 20-month compared to IDC-P(-) patients (HR = 2.28, 95% CI:1.35–3.86; 14.7 vs.34.5 months, *p* = 0.002) (Figure 1C and Supplementary Table 1). In addition, other parameters, including PSADT, GS, CFS, therapeutic strategies and pain score were also risk factors of OS (Supplementary Table 1).

Cox proportional hazard models were conducted to identify predictors of OS. Both univariate and multivariate analyses indicated that IDC-P status was one of the most significant predictors of OS. Notably, the addition of IDC-P status could dramatically improve the C-index of the basic model (0.756 vs. 0.777, *p* = 0.018, Table 2), confirming the powerful prognostic value of IDC-P in mCRPC.

**Table 2:** Multivariate analysis of OS for patients with mCRPC

	Univariate analysis		Multivariate analysis			
			Model without IDC-P status		Model with IDC-P status	
	HR (95% CI)	*p* value	HR (95% CI)	*p* value	HR (95% CI)	*p* value
CFS (mo), ≥ 10 vs. < 10	0.33 (0.20–0.56)	0.000	0.25 (0.14–0.45)	0.000	0.28 (0.15–0.50)	0.000
Gleason score, ≥ 8 vs. < 8	2.53 (1.01–6.34)	0.048	1.33 (0.52–3.42)	0.554	1.23 (0.48–3.16)	0.662
IDC-P status, IDC-P(+) vs. IDC-P(–)	2.28 (1.35–3.86)	0.002	–	–	1.91 (1.11–3.29)	0.020
Therapeutic scheme, 1st -line treatment vs. BSC	0.47(0.27–0.83)	0.009	0.51 (0.28–0.91)	0.023	0.50 (0.28–0.91)	0.022
ECOG score, ≥ 2 vs. < 2	2.56 (1.50–4.35)	0.001	3.65 (2.05–6.48)	0.000	3.68 (2.07–6.56)	0.000
ALP (IU/L), ≥ 160 vs < 160	2.82 (1.66–4.79)	0.000	1.86 (1.03–3.33)	0.038	1.91 (1.05–3.48)	0.034
LDH (IU/L), ≥ 250 vs < 250	2.50 (1.43–4.37)	0.001	1.87 (1.00–3.49)	0.049	1.85 (0.98–3.49)	0.058
C-index of the model	–	–	0.756	–	0.777	0.018*

*c-index test.

OS = overall survival from CRPC to death; HR = hazard ratio; CI = confidence interval; CFS = CRPC-free survival; IDC-P = intraductal carcinoma of the prostate; ECOG = Eastern Cooperative Oncology Group; BSC = best supportive care; ALP = alkaline phosphatase; LDH = lactate dehydrogenase.

### IDC-P had different impacts on the therapeutic efficacy of Doc or Abi

**Figure 2:**
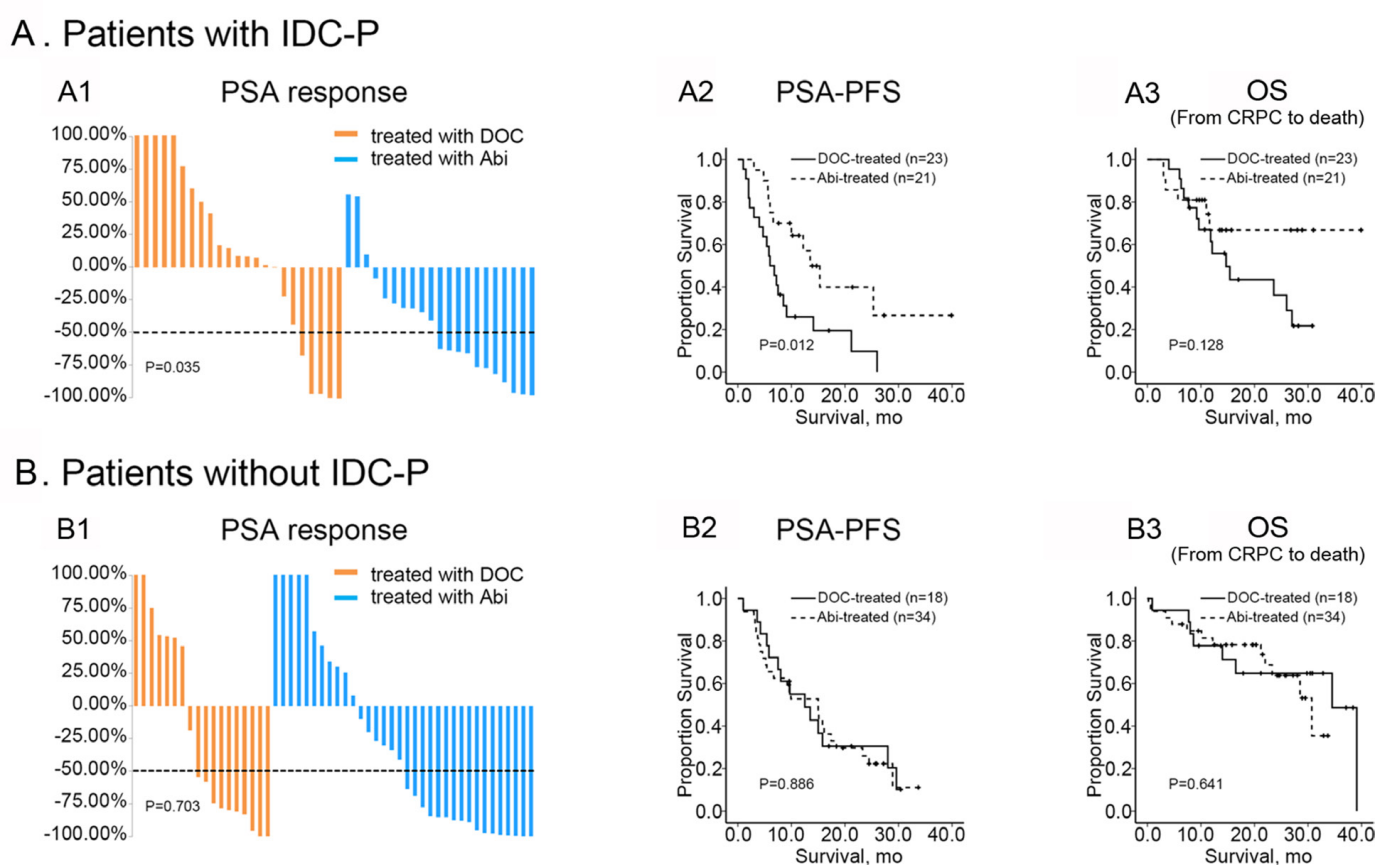
Therapeutic efficacy of Doc and Abi in patients with or without IDC-P (**A1, A2 and A3**) Comparison of PSA response, PSA-PFS and OS between IDC-P(+) patients treated with Abi or Doc; (**B1**, **B2** and **B3**) Comparison of PSA response, PSA-PFS and OS between IDC-P(–) patients treated with Abi or Doc.

Overall, 96/131(73.3%) patients received standard first-line therapies. Among them, 55 and 41 patients were treated with Abi or Doc, respectively. Men with first-line treatments obtained a significant survival benefit compared with those without standard therapies (*n* = 35), with a 15-month improvement in OS (30.7 vs. 15.8 months, *p* = 0.006). To explore the predictive role of IDC-P in different therapeutic schemes, men with or without IDC-P were further compared. In men without IDC-P, there appeared to be no difference in impact on PSA response between therapies, with both agents producing similar response rates (55.6% vs. 56.7%, *p* = 0.703, Figure 2B1 and Table 3). Of note, IDC-P(+) patients had a clear predisposition for unfavorable PSA response after receiving Doc, but not Abi (21.7% vs. 52.4%, *p* = 0.035, Figure 2A1 and Table 3). Moreover, in men with IDC-P, median PSA-PFS was significantly more prolonged in the Abi-treated group than in the Doc-treated group (13.5 vs. 6.0 months, *p* = 0.012). However, in those without IDC-P, such tendency could not be observed between these two treatment groups (Figure 2A2 and 2B2). Additionally, Abi could extend mean OS by nearly 9-month compared to Doc for IDC-P(+) patients, although this difference did not achieve statistical significance (*p* = 0.128) (Figure 2A3). No difference in OS was observed between Abi and Doc treated IDC-P(-) patients (Figure 2B3).

**Table 3:** Clinical outcomes in mCRPC patients with different IDC-P status

	Without IDC-P			With IDC-P		
	Docetaxel (*n* = 18)	Abiraterone (*n* = 34)	*p* value	Docetaxel (*n* = 23)	Abiraterone (*n* = 21)	*p* value
PSA response						
Continued PSA progression, *n* (%)	7 (38.9%)	11 (32.4%)	0.6371	16 (69.6%)	3 (14.3%)	< 0.001*
Decrease in PSA, *n* (%)	11 (61.1%)	23 (67.6%)	0.6371	7 (30.4%)	18 (85.7%)	< 0.001*
PSA response, *n* (%)	10 (55.6%)	17 (50.0%)	0.7031	5 (21.7%)	11 (52.4%)	0.035*
PSA-PFS, median (95% CI), (mo)	12.5 (5.2–19.8)	15.0 (7.7–22.3)	0.8862	6.0 (3.9–8.1)	13.5 (9.1–17.9)	0.012#
OS, median (95% CI), (mo)	34.5 (23.2–45.8)	30.7 (22.1–39.3)	0.6412	14.7 (8.7–20.7)	not reach	0.128#

*: Chi-square test; #: Log-rank test.

PSA response was defined as a PSA decline over 50% from the baseline.

OS = overall survival from CRPC to death; PSA = prostate specific antigen; PSA-PFS=PSA-progression free survival; IDC-P = intraductal carcinoma of the prostate; CI=confidence interval.

Cox regression further indicated that therapeutic scheme could have a remarkable power to predict disease progression in IDC-P(+) men. Notably, Abi was associated with prolonged time to PSA-PFS (HR = 0.33, 95% CI: 0.14–0.79, *p* = 0.013), and could significantly increase the predictive accuracy of the standard model, with an increase of the C-index from 0.719 to 0.778 (*p* = 0.009) (Table 4).

**Table 4:** PSA-PFS and OS for IDC-P-positive mCRPC patients

PSA-PFS	Univariate analysis		Model without therapeutic scheme		Model with therapeutic scheme	
	HR (95% CI)	*p* value	HR (95% CI)	*p* value	HR (95% CI)	*p* value
CFS (mo), ≥ 10 vs. < 10	0.55 (0.26–1.17)	0.119	0.72 (0.32–1.62)	0.432	0.52 (0.21–1.25)	0.144
Gleason score, ≥ 8 vs. < 8	1.72 (0.40–7.34)	0.466	2.50 (0.57–11.00)	0.226	1.76 (0.38–8.09)	0.467
Therapeutic scheme, Abi vs. Doc	0.40 (0.19–0.84)	0.015	–	–	0.33 (0.14–0.79)	0.013
ECOG score, ≥ 2 vs. < 2	1.60 (0.71–3.62)	0.259	2.16 (0.92–5.04)	0.076	2.00 (0.81–4.91)	0.132
ALP (IU/L), ≥ 160 vs < 160	2.97 (1.36–6.49)	0.006	2.10 (0.81–5.42)	0.125	2.82 (1.02–7.83)	0.046
LDH (IU/L), ≥ 250 vs < 250	3.20 (1.44–7.13)	0.004	2.63 (1.02–6.79)	0.046	1.68 (0.59–4.77)	0.334
C-index of the model	-	-	0.719	-	0.778	0.009*
**OS**	**Univariate analysis**		**Model without therapeutic scheme**		**Model with therapeutic scheme**	
	**HR (95% CI)**	***p*** **value**	**HR (95% CI)**	***p*** **value**	**HR (95% CI)**	***p*** **value**
CFS (mo), ≥ 10 vs. < 10	0.25 (0.09–0.66)	0.005	0.15 (0.04–0.50)	0.002	0.14 (0.04–0.46)	0.001
Gleason score, ≥ 8 vs. < 8	1.03 (0.24–4.45)	0.969	1.50 (0.33–6.86)	0.604	1.20 (0.25–5.79)	0.819
Therapeutic scheme, Abi vs. Doc	0.48 (0.19–1.26)	0.137	–	–	0.44 (0.14–1.37)	0.157
ECOG score, ≥ 2 vs. < 2	1.69 (0.64–4.42)	0.288	2.65 (0.89–7.94)	0.081	2.18 (0.71–6.73)	0.174
ALP (IU/L), ≥ 160 vs < 160	4.92 (1.84–13.17)	0.002	2.13 (0.61–7.42)	0.233	3.00 (0.73–12.26)	0.127
LDH (IU/L), ≥ 250 vs < 250	4.87 (1.79–13.26)	0.002	3.37 (0.89–12.79)	0.075	2.37 (0.55–10.34)	0.250
C-index of the model	-	-	0.829		0.823	0.438*

*c-index test.

OS = overall survival from CRPC to death; HR = hazard ratio; CI = confidence interval; CFS = CRPC-free survival; IDC-P = intraductal carcinoma of the prostate; ECOG = Eastern Cooperative Oncology Group; Doc = docetaxel; Abi = abiraterone; ALP = alkaline phosphatase; LDH = lactate dehydrogenase.

In our previous study, subtypes of IDC-P seemed to further impact therapeutic efficacy in mCRPC patients.[15] Therefore, IDC-P(+) patients were further sub-classified and analyzed according to different subtypes. Among 44 patients with IDC-P who received standard treatment, 29.5% (13/44) were pure cribriform pattern while the other 70.5% (31/44) consisted of either solid or mixed patterns (with or without comedonecrosis) and were defined as non-pure cribriform pattern. Interestingly, men with pure cribriform pattern had similar PSA response rates to either Abi or Doc (Table 5). However, patients with non-pure cribriform pattern had a significantly reduced PSA response rate to Doc compared to the PSA response rate when treated with Abi (1/16, 6.3% vs. 7/15, 46.7%, *p* = 0.015, Table 5). This finding suggested that the poor sensitivity of IDC-P(+) patients to Doc might be further related to different subtypes of IDC-P.

**Table 5:** PSA response in mCRPC patients with different IDC-P subtypes

	Pure Cribriform			Non-pure Cribriform		
	Doc(*n* = 7)	Abi(*n* = 6)	*p* value	Doc(*n* = 16)	Abi(*n* = 15)	*p* value
Continued PSA progression, *n* (%)	2 (28.6%)	0 (0.0%)	0.4621	14 (87.5%)	3 (20.0%)	< 0.001^1^
Decrease in PSA, *n* (%)	5 (71.4%)	6 (100.0%)	0.4621	2 (12.5%)	12 (80.0%)	< 0.001^1^
PSA response, *n* (%)	4 (57.1%)	4 (66.7%)	1.0001	1 (6.3%)	7 (46.7%)	0.015^1^

1: Fisher exact test.

PSA response was defined as a PSA decline over 50% from the baseline.

IDC-P = intraductal carcinoma of the prostate; PSA = prostate specific antigen; Doc = docetaxel; Abi = abiraterone.

## DISCUSSION

In the current study, we showed that IDC-P in men with mCRPC was associated with faster disease progression and shorter OS than those without IDC-P. We further demonstrated that Abi might have superiority to Doc in patients with IDC-P, especially non-pure cribriform pattern. Therefore, the totality of our data suggested that IDC-P might not only represent a prognostic harbinger, but also a predictive marker aiding therapeutic selection in mCRPC.

Since 2010, Abi and Enzalutamide have been sequentially approved by the FDA as standard first-line therapeutics for mCRPC [4–6]. These novel agents, together with Doc, provide more opportunities and choices for treatment of this intractable disease. However, some men do not benefit from these agents. A key issue is to determine how to select optimal treatment regimens to achieve optimized personalized therapy. Unfortunately, predictive biomarkers that can guide optimal treatment choices for patients are still limited. In 2014, the association of AR-V7 with Abi- or Enzalutamide-resistance in men with mCRPC was first reported [9]. Yet, recently, Bernemann and his colleagues questioned and challenged the predictive value of AR-V7 [11]. Besides, the detection of CTCs, which can be subsequently probed for AR-V7 expression, relies on specific cell surface antigens. Due to the high heterogeneity of tumor cells, inconclusive results are sometimes unavoidable [16].

IDC-P was firstly described by Kovi et al. in 1985, [17] but recently, interest in its role in the progression of PCa has increased. Several studies have initially looked at the prognostic significance of IDC-P in patients with localized PCa (Supplementary Table 2). Linderberg identified the area in the prostate that gave rise to metastases by exome sequencing, searching for clonal relationship between multiple primary tumors and metastatic lesions. They discovered that IDC-P in the primary tumor was the potential origin for distal metastasis [18]. The latest studies reported that BRCA2 mutations coupled with PTEN loss were associated with the evolution of IDC-P [19, 20].

In the past 5 years, our group has focused on the association of IDC-P with metastatic PCa. Our previous data showed that IDC-P was associated with rapid occurrence of mCRPC, adverse outcome and resistance to chemotherapy in patients with metastatic disease, and that detection of IDC-P dramatically increased with disease progression [14, 15]. In this study, we further investigate the association between IDC-P and clinical outcome in mCRPC patients. Here, we demonstrate that when compared to IDC-P(-) patients, IDC-P(+) patients have a more rapid PSADT and nearly 20-month shorter median OS. After adjusting for treatment selection, baseline patient characteristics and pathological variables, IDC-P retained its prognostic efficacy. Taken together, our current and previous findings suggest that IDC-P should be recognized as a critical risk factor in predicting clinical outcome throughout all stages of PCa, and adequate attention should be paid to the precise diagnosis of IDC-P in PCa patients.

Another important finding of this study is that IDC-P could be a potential marker in predicting insensitivity to Doc-based chemotherapy. This could help physicians make decisions to select optimal first-line therapeutic regimens. Unlike so called “liquid biopsies”, the diagnosis of IDC-P relies on available tissues obtained from the tumor. However false-negative diagnosis is possible. Improvement of biopsy technique may increase the predictive value of IDC-P for mCRPC patients.

At the time of re-biopsy to confirm mCRPC, almost half patients (47.3%) had detectable IDC-P. Importantly, among patients receiving Doc, there was a significant difference in PSA response, PSA-PFS and OS (from mCRPC to death) according to IDC-P status. Subanalysis according to IDC-P patterns further confirmed that the poor sensitivity of IDC-P(+) patients to Doc was mainly from non-pure cribriform pattern. Multivariate analysis and C-index suggested IDC-P status could be a powerful predictive factor aiding the selection of optimal therapeutic regimens as first-line therapy. Till now, evidence has supported the predictive role of AR-V7 in precluding Abi or Enzalutimade as first-line therapy for mCRPC, [9] while our result observed the promising predictive role of IDC-P in precluding Doc as first-line therapy. Future work may lead to optimal and personalized therapy based on combined detection of AR-V7 status from “liquid biopsy” and IDC-P status from “tissue biopsy”.

In contrast to other studies from more developed countries, a majority of patients (88/96, 91.7%) in the present study seemed to only be able to get one standard therapy for mCRPC mainly due to the heavy socioeconomic burden. Undoubtedly, for these patients, it is of crucial importance to select optimal first-line treatment.

Several limitations exist in our study. Due to a relative small-size retrospective study with data from a single medical center, selection bias cannot be ruled out. Besides, because of non-persistently performed of radiological examinations, radiological changes could not be evaluated for every patient enrolled in this study. Importantly, exact mechanisms of primary resistance to Doc associated with IDC-P are unknown, and still remain to be elucidated.

In conclusion, first-line treatment options for mCRPC are increasing but optimal strategies for each patient are unknown. Additionally, treatment-guiding markers are few and poorly understood. We found that the presence of IDC-P easily obtained from needle biopsy is an independent prognosticator for clinical outcome in mCRPC patients. This result along with our previous work demonstrates the potential importance in assessing IDC-P status at every stage of PCa. Interestingly, IDC-P, especially the non-pure cribriform pattern, appeared to be associated with much poorer response to Doc than Abi in mCRPC patients. IDC-P status should be considered as a novel predictive marker helping physicians preclude Doc as first-line therapy. Future work to understand the mechanisms driving these altered therapeutic responses will improve our understanding of this prognosticator. Additional efforts using larger-scale studies will aid in validation of our findings.

## MATERIALS AND METHODS

### Study design

From 2009 to 2016, a total of 131 patients with initially diagnosed bone metastatic PCa who had progressed to mCRPC in West China Hospital were enrolled. After signing informed consents, all patients received prostate biopsies at the time of initial diagnosis and the confirmation of mCRPC, respectively. Both biopsies were 12-core ultrasound-guided transperineal prostate biopsy. Those who did not have the repeat biopsy were not included in this study. With the exception of IDC-P, patients with other non-acinar adenocarcinoma including ductal adenocarcinoma, neuroendocrine carcinoma or small cell carcinoma of the prostate were excluded. All patients received maximal androgen blockade (MAB) from the initial diagnosis of PCa, which was surgical or medical castration combined with antiandrogens. The median time from initial diagnosis to mCRPC (CRPC-free survival, CFS) was 13.7 months. Within 4–6 weeks of mCRPC confirmation, a majority of patients (96/131, 73.3%) received either abiraterone (Abi) plus prednisone (*n* = 55) or Doc plus prednisone (*n* = 41) as first-line treatment. The remaining patients received best supportive care (BSC) (35/131, 26.7%) as therapy due to either financial hardship or fear of drugs adverse events. The median treatment duration of Abi and Doc was 6.8 and 8.8 cycles, respectively. After disease progression, only a few patients (*n* = 8) received sequential treatment, mainly owing to financial hardship. The cut-off point for analysis was Aug 1, 2016.

### Outcomes

Baseline characteristics of 131 patients were collected at the time of mCRPC, including IDC-P status, age, CFS, castration type, visceral metastasis, ECOG score, pain score, number of bone scan lesions, PSA doubling time (PSADT), serum prostate specific antigen (PSA) level, serum testosterone level, serum hemoglobin (HGB) level, serum lactate dehydrogenase (LDH) level and serum ALP level. Owing to the endocrine response caused by androgen deprivation therapy (ADT), Gleason score (GS) at re-biopsy couldn't be evaluated accurately. Therefore, GS at the initial biopsy was used for analysis. All the biopsy specimens were successively processed as following steps: fixed in 10% buffered formalin, paraffin-embedded, cut at 4-mm thickness, and stained with hematoxylin and eosin. IDC-P status was reviewed and reported by two independent urological pathologists according to Epstein's criteria, which is malignant epithelial cells filling large acini and prostatic ducts, with preservation of basal cells forming either: (1) solid or dense cribriform patterns or; (2) loose cribriform or micropapillary patterns with either marked nuclear atypia (nuclear size 6 × normal or larger) or comedonecrosis. [21] The pathological images of the positive cases could be referred to our previous study [15]. mCRPC was defined according to 2014 EAU guidelines, i.e., three consecutive rises in serum PSA, occurring 1 week apart, resulting in two 50% increases over the nadir, with a PSA> 2 ng/ml, despite a castration testosterone level (< 0.5 ng/ml) [22]. PSADT was calculated according to the MSKCC online nomogram by using the first three PSA measurements after mCRPC [23].

The primary endpoint was PSA response (≥ 50% decline in PSA level from baseline, maintained for ≥ 4 weeks) and PSA-PFS. PSA-PFS was defined as an increase in the PSA level of 25% or more above the nadir (and by ≥ 2 ng/ml), with confirmation 4 or more weeks later according to PCWG2 criteria. [24] Secondary end point was OS defined as the time from the initiation of therapy after mCRPC to death from any cause.

### Statistics

Mann-Whitney *U* test, chi-square test, and Fisher exact test were used to determine the statistical significance among different variables (SPSS 20.0). PSA-PFS and OS were assessed by Kaplan-Meier curves, while differences between the survival curves were compared by Log-rank test. Multivariable analyses were adjusted for CFS, GS, Eastern Cooperative Oncology Group (ECOG), ALP, LDH, therapeutic scheme and IDC-P status. To avoid multicollinearity and overfitting of models, factors including PSADT, pain score, bone scan lesions, baseline testosterone and baseline PSA were not included. Harrell's C-index was used to further assess the discrimination of models (R software). A *p* < 0.05 was defined as statistical significance.

## SUPPLEMENTARY MATERIALS FIGURES



## Abbreviations

Abi: abiraterone
ADT: androgen deprivation therapy
ALP: alkaline phosphatase
AR-V7: androgen receptor variant 7
BSC: best supportive care
CFS: CRPC-free survival
Doc: docetaxel
ECOG: Eastern Cooperative Oncology Group
GS: Gleason score
HGB: hemoglobin
IDC-P: intraductal carcinoma of the prostate
LDH: lactate dehydrogenase
MAB: maximal androgen blockade
mCRPC: metastatic castration-resistant prostate cancer
OS: overall survival
PCa: prostate cancer
PFS: progression free survival
PSA: prostate specific antigen
PSADT: PSA doubling time

## References

[1] National Comprehensive Cancer Network NCCN clinical practice guidelines in oncology (NCCN Guidelines). http://www.nccn.org/professionals/physician_gls/pdf/prostate.pdf.

[2] Petrylak DP, Tangen CM, Hussain MH, Lara PN, Jones JA, Taplin ME, Burch PA, Berry D, Moinpour C, Kohli M, Benson MC, Small EJ, Raghavan D (2004). Docetaxel and estramustine compared with mitoxantrone and prednisone for advanced refractory prostate cancer. N Engl J Med.

[3] Tannock IF, de Wit R, Berry WR, Horti J, Pluzanska A, Chi KN, Oudard S, Théodore C, James ND, Turesson I, Rosenthal MA, Eisenberger MA (2004). Docetaxel plus prednisone or mitoxantrone plus prednisone for advanced prostate cancer. N Engl J Med.

[4] Fizazi K, Scher HI, Molina A, Logothetis CJ, Chi KN, Jones RJ, Staffurth JN, North S, Vogelzang NJ, Saad F, Mainwaring P, Harland S, Goodman OB (2012). Abiraterone acetate for treatment of metastatic castration-resistant prostate cancer: final overall survival analysis of the COU-AA-301 randomised, double-blind, placebo-controlled phase 3 study. Lancet Oncol.

[5] Ryan CJ, Smith MR, Fizazi K, Saad F, Mulders PF, Sternberg CN, Miller K, Logothetis CJ, Shore ND, Small EJ, Carles J, Flaig TW, Taplin ME (2015). Abiraterone acetate plus prednisone versus placebo plus prednisone in chemotherapy-naive men with metastatic castration-resistant prostate cancer (COU-AA-302): final overall survival analysis of a randomised, double-blind, placebo-controlled phase 3 study. Lancet Oncol.

[6] Beer TM, Armstrong AJ, Rathkopf DE, Loriot Y, Sternberg CN, Higano CS, Iversen P, Bhattacharya S, Carles J, Chowdhury S, Davis ID, de Bono JS, Evans CP (2014). Enzalutamide in metastatic prostate cancer before chemotherapy. N Engl J Med.

[7] Karantanos T, Evans CP, Tombal B, Thompson TC, Montironi R, Isaacs WB (2015). Understanding the mechanisms of androgen deprivation resistance in prostate cancer at the molecular level. Eur Urol.

[8] Armstrong CM, Gao AC (2015). Drug resistance in castration resistant prostate cancer: resistance mechanisms and emerging treatment strategies. Am J Clin Exp Urol.

[9] Antonarakis ES, Lu C, Wang H, Luber B, Nakazawa M, Roeser JC, Chen Y, Mohammad TA, Chen Y, Fedor HL, Lotan TL, Zheng Q, De Marzo AM (2014). AR-V7 and Resistance to Enzalutamide and Abiraterone in Prostate Cancer. N Engl J Med.

[10] Antonarakis ES, Lu C, Luber B, Wang H, Chen Y, Nakazawa M, Nadal R, Paller CJ, Denmeade SR, Carducci MA, Eisenberger MA, Luo J (2015). Androgen Receptor Splice Variant 7 and Efficacy of Taxane Chemotherapy in Patients With Metastatic Castration-Resistant Prostate Cancer. JAMA Oncol.

[11] Bernemann C, Schnoeller TJ, Luedeke M, Steinestel K, Boegemann M, Schrader AJ, Steinestel J Expression of AR-V7 in Circulating Tumour Cells Does Not Preclude Response to Next Generation Androgen Deprivation Therapy in Patients with Castration Resistant Prostate Cancer. Eur Urol.

[12] Pickup M, Van der Kwast TH (2007). My approach to intraductal lesions of the prostate gland. J Clin Pathol.

[13] Humphrey PA, Moch H, Cubilla AL, Ulbright TM, Reuter VE (2016). The 2016 WHO Classification of Tumours of the Urinary System and Male Genital Organs-Part B: Prostate and Bladder Tumours. Eur Urol.

[14] Zhao T, Liao B, Yao J, Liu J, Huang R, Shen P, Peng Z, Gui H, Chen X, Zhang P, Zhu Y, Li X, Wei Q (2015). Is there any prognostic impact of intraductal carcinoma of prostate in initial diagnosed aggressively metastatic prostate cancer?. Prostate.

[15] Chen Z, Chen N, Shen P, Gong J, Li X, Zhao T, Liao B, Liu L, Liu Z, Zhang X, Liu J, Peng Z, Chen X (2015). The presence and clinical implication of intraductal carcinoma of prostate in metastatic castration resistant prostate cancer. Prostate.

[16] Alix-Panabières C, Pantel K (2014). Challenges in circulating tumour cell research. Nat Rev Cancer.

[17] Kovi J, Jackson MA, Heshmat MY (1985). Ductal spread in prostatic carcinoma. Cancer.

[18] Lindberg J, Kristiansen A, Wiklund P, Grönberg H, Egevad L (2015). Tracking the origin of metastatic prostate cancer. Eur Urol.

[19] Schneider TM, Osunkoya AO (2014). ERG expression in intraductal carcinoma of the prostate: comparison with adjacent invasive prostatic adenocarcinoma. Mod Pathol.

[20] Risbridger GP, Taylor RA, Clouston D, Sliwinski A, Thorne H, Hunter S, Li J, Mitchell G, Murphy D, Frydenberg M, Pook D, Pedersen J, Toivanen R (2015). Patient-derived xenografts reveal that intraductal carcinoma of the prostate is a prominent pathology in BRCA2 mutation carriers with prostate cancer and correlates with poor prognosis. Eur Urol.

[21] Guo CC, Epstein JI (2006). Intraductal carcinoma of the prostate on needle biopsy: Histologic features and clinical significance. Mod Pathol.

[22] Heidenreich A, Bastian PJ, Bellmunt J, Bolla M, Joniau S, van der Kwast T, Mason M, Matveev V, Wiegel T, Zattoni F, Mottet N, EAU guidelines on prostate cancer (2014). Part II: Treatment of advanced, relapsing, and castration-resistant prostate cancer. Eur Urol.

[23] Memorial Sloan Kettering Cancer Center Prostate cancer nomograms: PSA doubling time. http://www.mskcc.org/applications/nomograms/prostate/PsaDoublingTime.aspx.

[24] Scher HI, Halabi S, Tannock I, Morris M, Sternberg CN, Carducci MA, Eisenberger MA, Higano C, Bubley GJ, Dreicer R, Petrylak D, Kantoff P, Basch E (2008). Design and end points of clinical trials for patients with progressive prostate cancer and castrate levels of testosterone: recommendations of the Prostate Cancer Clinical Trials Working Group. J Clin Oncol.

