# Supplementary material for: The prognostic implication of intraductal carcinoma of the prostate in metastatic castration-resistant prostate cancer and its potential predictive value in those treated with docetaxel or abiraterone as first-line therapy
Source: Oncotarget. 2017 Jul 24;8(33):55374–83. doi: 10.18632/oncotarget.19520 (PMC5589665; doi:10.18632/oncotarget.19520)
Supplement: Supplementary file 1 [file oncotarget-08-55374-s001.pdf]

The prognostic implication of intraductal carcinoma of the prostate in metastatic castration-resistant prostate cancer and its potential predictive value in those treated with docetaxel or abiraterone as first-line therapy

SUPPLEMENTARY MATERIALS

Supplementary Table 1: Log-rank analysis of the predictors for OS in patients with mCRPC

		OS		
	n (%)	Median	95% CI	*p* value*
IDC-P status				
IDC-P(+)	62 (47.3%)	14.7	9.28–20.12	0.002
IDC-P(–)	69 (52.7%)	34.5	26.56–42.44	
Age (y)				
≥ 75	95 (72.5%)	25.12	21.32–28.92	0.576
< 75	36 (27.5%)	22	10.19–33.82	
CFS (mo)				
≥ 10	85 (64.9%)	34.5	26.74–42.26	< 0.001
< 10	46 (35.1%)	10.1	7.15–13.05	
Treatment				
1st treatment	96 (73.3%)	30.7	24.30–37.10	0.007
BSC and others	35 (26.7%)	15.8	6.24–25.36	
Gleason score				
< 8	21 (16.0%)	27.3	22.4–32.2	0.040
8–10	110 (84.0%)	22.3	19.2–25.3	
ECOG score				
≥ 2	100 (76.3%)	15.8	8.84–22.76	< 0.001
< 2	31 (23.7%)	34.5	26.74–42.26	
Pain score				
> 3	45 (34.4%)	9.5	.68–11.32	< 0.001
≤ 3	86 (65.6%)	39.1	26.07–52.13	
Bone Scan Lesions				
≥ 10	84 (64.1%)	17.5	10.47–24.53	0.041
< 10	47 (35.9%)	30.7	23.98–37.42	
PSADT (days)				
≥ 30	95 (72.5%)	30.7	22.45–38.95	0.012
< 30	36 (27.5%)	12.2	7.77–16.63	
Testosterone (ng/mL)				
≥ 0.09	71 (54.2%)	28.5	19.97–37.04	0.847
< 0.09	60 (45.8%)	22	11.36–32.64	
HGB (g/L)				
< 120	33 (25.2%)	16.5	0.00–33.98	0.174
≥ 120	98 (74.8%)	30.7	22.13–39.27	
LDH (IU/L)				
≥ 250	71 (54.2%)	16.8	11.32–22.28	0.001
< 250	60 (45.8%)	39.1	27.08–51.12	
ALP (IU/L)				
≥ 160	58 (44.3%)	11.6	5.42–17.78	< 0.001
< 160	73 (55.7%)	34.5	24.40–44.60	

*Log-rank test.
CRPC = castration-resistant prostate cancer; CFS = CRPC free survival; IDC-P = intraductal carcinoma of the prostate; BSC = best supportive care; ECOG = Eastern Cooperative Oncology Group; PSA = prostate specific antigen; PSADT= prostate specific antigen doubling time; HGB = hemoglobin; LDH = lactate dehydrogenase; ALP = alkaline phosphatase.

Supplementary Table 2: Summary of studies about the prognostic role of IDC-P in patients with prostate cancer

Study	Country	Pathological Specimens	Patients	*N*	Main conclusion
Cohen RJ 1998 [25]	New Zealand	Prostate biopsy specimens	Localized PCa	59	IDC-P was an independent variable that significantly improved the prediction of pathological stage and tumor volume, and furthermore, was closely related to ($r = 0.53$, $p = 0.001$) and accurately predicted treatment failure.
O'Brien C 2010 [26]	Portland	RP specimens	High risk PCa	57	In both univariate analyses ($p = 0.001$) and multivariate analyses ($p = 0.007$), IDC-P was associated with shorter relapse-free survival.
Efstathiou E 2010 [27]	America	RP specimens	High risk PCa	115	The presence of cribriform or intraductal spread morphology was a strong predictor of biochemical relapse (Relative risk = 2.98, $p < 0.001$).
Van der Kwast T 2012 [28]	Canada and Belgium	Prostate biopsy specimens	Intermediate or high risk PCa	250	IDC-P was a strong prognosticator for early (< 36 months) biochemical relapse (HR, 95% CI: 7.3, 1.7–30.4; $p = 0.007$) in patients with intermediate risk PCa.<br>IDC-P was a strong prognosticator for clinical disease-free survival in patients with high risk PCa treated with radiotherapy (HR, 95% CI: 3.54, 1.88–6.69; $p < 0.0001$) or radiotherapy plus long-term androgen deprivation (HR, 95% CI: 2.83, 1.16–6.92; $p = 0.0018$).
Miyai K 2014 [29]	America	RP specimens	localized PCa	901	IDC-P was a marker of adverse pathologic features and clinical aggressiveness. Patients with IDC-P were with shorter biochemical recurrence than those with high-grade prostatic intraepithelial neoplasia (Log-rank test, $p < 0.0001$) or atypical cribriform lesion (Log-rank test, $p < 0.022$).
Trudel D 2014 [30]	Canada	RP specimens	localized PCa	246	The presence of any amount of large cribriform or IDC-P had a highly significant prognostic effect on biochemical recurrence-free rate (HR, 95% CI: 2.98, 1.68–5.28, $p = 0.0002$) after adjusting for GS, surgical margin status and pathological stage.
Kimura K 2015 [31]	Japan	RP specimens	High risk PCa	206	IDC-P in RP specimens was an independent risk factor for progression-free survival (HR, 95%CI: 3.07, 1.44–6.58, $p = 0.0038$) and cancer-specific survival (HR, 95% CI: 4.48, 1.22–16.41, 0.0238).
Zhao T 2015 [14]	China	Prostate biopsy specimens	Metastatic PCa	278	The presence of IDC-P was not only an independent prognostic factor predicting<br>shorter time of CRPC (HR = 4.031, $P = 0.035$), but also for poorer OS (HR = 2.499, $P = 0.006$)
Chen Z 2015 [15]	China	Prostate biopsy specimens	mCRPC	45	IDC-P was significantly associated with rapid disease progression. 13/28 (46.4%) CRPC patients with IDC-P had PSADT less than 30 days, while, only 1/17 (5.9%) patient without IDC-P had a less than 30 days PSADT (X2 = 8.114, $P = 0.004$).<br>Among patients treated with docetaxel ($n = 24$), those with IDC-P showed more unfavorable response than those without IDC-P (20% vs. 66.7%, $P = 0.022$).
Kato M 2016 [32]	Japan	Prostate biopsy specimens	metastatic PCa	150	The presence of IDC-P was a significant prognostic parameter for cancer-specific survival (HR, 95% CI: 2.13, 1.14–3.99, $p = 0.0181$) and overall survival (HR, 95% CI: 2.66, 1.47–4.79, $p = 0.0012$) in PCa patients with distant metastasis at presentation.
Saeter T 2017 [33]	Norway	Prostate biopsy specimens	PCa (M0 or Mx)	283	IDC-P on diagnostic needle biopsy is an indicator of prostate cancer with a high risk of mortality. (HR, 95% CI: 3.3, 2.0–5.7, $p < 0.001$ in univariate analysis; HR, 95%CI: 1.7, 0.9–2.8, $p = 0.07$ in multivariate analysis)
Kweldam CF 2017 [34]	Nederland	Prostate biopsy specimens	localized PCa	486	The presence of cribriform and intraductal growth in prostate cancer, is a strong clinical prognostic marker with poorer OS (HR, 95% CI: 2,6, 1.4–4.8, $p = 0.002$)

RP = Radical prostatectomy; N = sample size; PCa = Prostate cancer; mCRPC = metastatic castration-resistant prostate cancer; HR=hazard ratio; CI = confidence interval. GS = Gleason score; OS = overall survival; PSADT = PSA-doubling time.
